# Histone profiling reveals the H1.3 histone variant as a prognostic biomarker for pancreatic ductal adenocarcinoma

**DOI:** 10.1186/s12885-017-3834-z

**Published:** 2017-12-02

**Authors:** Monika Bauden, Theresa Kristl, Agata Sasor, Bodil Andersson, György Marko-Varga, Roland Andersson, Daniel Ansari

**Affiliations:** 1 Department of Surgery, Clinical Sciences Lund, Lund University, Skåne University Hospital, SE-221 85 Lund, Sweden; 2 Clinical Protein Science & Imaging, Department of Biomedical Engineering, Lund University, Biomedical Center, Lund, Sweden; 3 Department of Pathology, Skåne University Hospital, Lund, Sweden

**Keywords:** Biomarkers, Epigenetics, Histone variants, H1.3, LC-MS/MS, Immunohistochemistry, Pancreatic Ductal Adenocarcinoma

## Abstract

**Background:**

Epigenetic alterations have been recognized as important contributors to the pathogenesis of PDAC. However, the role of histone variants in pancreatic tumor progression is still not completely understood. The aim of this study was to explore the expression and prognostic significance of histone protein variants in PDAC patients.

**Methods:**

Liquid chromatography-tandem mass spectrometry (LC-MS/MS) was employed for qualitative analysis of histone variants and histone related post-translational modifications (PTMs) in PDAC and normal pancreatic tissues. Survival analysis was conducted using the Kaplan-Meier method and Cox proportional hazards regression.

**Results:**

Histone variant H1.3 was found to be differentially expressed (*p* = 0.005) and was selected as a PDAC specific histone variant candidate. The prognostic role of H1.3 was evaluated in an external cohort of patients with resected PDAC using immunohistochemistry. Intratumor expression of H1.3 was found to be an important risk factor for overall survival in PDAC, with an adjusted HR value of 2.6 (95% CI 1.1–6.1), *p* = 0.029.

**Conclusion:**

We suggest that the intratumor histone H1.3 expression as reported herein, may serve as a new epigenetic biomarker for PDAC.

## Background

Pancreatic ductal adenocarcinoma (PDAC) is the most frequent histologic subtype of pancreatic cancer and accounts for one of the most aggressive malignancies. With an extremely low five-year survival rate, PDAC represents the fourth leading cause of cancer-related deaths in the United States and Europe [1, 2]. At the time of diagnosis, most patients have developed a locally advanced or metastatic disease, which limits the possibilities for therapeutic intervention and contributes to the poor prognosis [3, 4]. Detailed understanding of the biology behind pancreatic cancer is crucial for the improvement of clinical outcome as well as for the discovery of new biomarkers for early diagnosis, prognosis, and therapeutic targeting.

It is now apparent that, besides the extensive genetic alterations, an aberrant epigenetic regulation, including modifications of the chromatin structure, also significantly contributes to the pathogenesis of PDAC [5–7]. Chromatin depositions of histone variants have been implicated in the establishment and maintenance of the epigenetic states. Variants of histone proteins are further involved in fundamental cellular processes, such as regulation of transcriptional activity or DNA repair, hence considered as contributors to tumor progression [8, 9].

Histone proteins constitute nucleosomes, which are the basic structural and functional components of the chromatin. The nucleosomes consist of superhelical DNA wrapped around a histone octamer composed of two copies of each histone protein H2A, H2B, H3 and H4. The higher order chromatin stabilization is facilitated by the linker histone H1 variants [10].

Histone proteins are usually divided into conventional, canonical histones that function mainly in the packaging of the newly replicated DNA and histone variants that replace the canonical histones when nucleosomes are disrupted, at any phase of the cell cycle. Histone isoforms within each histone family are distinguished from each other by a specific primary amino acid sequence, differing often with only a few amino acids. The incorporation of diverse histone variants can influence the functional properties of the nucleosome and thus affect the chromatin conformation and the accessibility of the genome. The nucleosomal assembly of histone variants, together with histone related post-translational modifications, is essential for the transition between active and silent chromatin states and thus plays a significant role in the epigenetic regulation of gene transcription [11–13]. Alteration of epigenetic processes involved in chromatin dynamics may ultimately promote cancer development and tumor progression [14, 15]. The availability of biobank materials and advanced proteomic analysis tools makes it possible to investigate the changes in chromatin-related epigenetics, including histone variants coinciding with the malignant transformation [16, 17]. The profile of histone variants, as the regulators of chromatin, should therefore be explored in order to provide further insights regarding PDAC pathobiology and guide new approaches for disease management to ameliorate the poor prognosis of PDAC.

Here, we report the profile of histone protein variants in PDAC tissue in relation to normal pancreas, assessed by high-resolution nano-liquid chromatography-tandem mass spectrometry (LC-MS/MS). The intratumor distribution of the PDAC specific histone variant candidate was verified by immunohistochemistry (IHC). The prognostic value of H1.3 expression in PDAC was explored using survival analysis.

## Methods

### Materials

Unless stated otherwise, the following chemicals and solvents were purchased from Sigma-Aldrich St. Louis, MO, USA; Tris-HCl, guanidine-HCl, ammonium bicarbonate (AMBIC), dithiothreitol (DTT), iodoacetamide (IAA), formic acid (FA), acetonitrile (ACN), sodium chloride (NaCl), sodium citrate, Tween 20, Triton X-100 and bovine serum albumin (BSA). Xylene, Pertex and hematoxylin were obtained from Histolab Products AB, Gothenburg, Sweden and ethanol (EtOH) from Solveco, Rosenberg, Sweden. Protein determination assay, peptide determination kit and Pierce LC-MS grade water was obtained from Thermo Scientific, Rockford, IL, USA. Milli-Q water was produced using an in-house installed purification system Q-POD Millipore (EMD Millipore, Billerica, MA, USA). Thermo-Fisher Scientific, Bremen, Germany was the supplier of analytical instruments used in this study, including EASY-nLC™ 1000 nanoflow liquid chromatography system and Q Exactive™ Plus Hybrid Quadrupole-Orbitrap™ mass spectrometer equipped with a Thermo Scientific™ EASY-Spray™ source. The table centrifuge 5415R, speed vacuum concentrator plus, and thermomixer Comfort were provided by Eppendorf AG, Hamburg, Germany.

### Analysis of the histone profile in PDAC using LC-MS/MS

#### Tissue acquisition

Fresh frozen PDAC tissue (*n* = 10) used for LC-MS/MS analysis was acquired from patients undergoing pancreaticoduodenectomy between July 2013 and April 2015 at the department of Surgery, Skåne University Hospital in Lund, Sweden. PDAC specimens were selected retrospectively from a local biobank, using information recorded in the hospital patient registry to obtain a study population as homogeneous as possible. The inclusion criteria were based on following parameters: histopathological diagnosis of low to moderately differentiated PDAC with a primary tumor located in the pancreatic head, stage T3 N1 (AJCC, 7th edition), no diabetes mellitus and no neoadjuvant therapy undertaken. The accepted co-morbidity was limited to cardiovascular associated disease, kidney stone and age-related conditions as e.g. benign prostate hyperplasia.

Fresh frozen pancreatic head biopsies (*n* = 10) were obtained from organ donors and acquired through the Lund University Diabetes Center (LUDC), a part of the national consortium Excellence of Diabetes Research in Sweden (EXODIAB) and co-analyzed as comparative healthy control.

#### Tissue processing

Respective fresh frozen specimens were separately pulverized in liquid N_2_ using dry ice chilled mortar and pestle and homogenized in extraction buffer (500 mM Tris-Cl, [pH 8] and 6 M guanidine-HCl in 50 mM AMBIC), supplemented with protease and phosphatase inhibitor. The crude homogenates were then subjected to four thaws and freeze cycles, followed by ultrasonic bath treatment for 20 min on ice and a short centrifugation to remove debris. The soluble proteins in the supernatant were reduced with 15 mM DTT for 60 min at 60 °C, alkylated for 30 min at room temperature (RT) using 50 mM IAA and precipitated overnight with ice cold absolute ethanol, with the ratio of one part sample and nine parts 99.5% EtOH. The precipitated proteins were dissolved in 50 mM AMBIC and quantified using the BCA assay. To increase the sequence coverage and the probability to identify the highly divergent subtypes among the conserved histone families, 130 μg of the precipitated protein fraction from respective tissue sample digested overnight at 37 °C using either Mass Spec Grade Trypsin/Lys-C Mix or Sequencing grade Glu-C (both from Promega, Madison, WI, USA), at a final protein enzyme ratio of 1:100. The next day, the digests were evaporated using a SpeedVac and dissolved in 50 μl mobile phase A (0.1% FA). The peptides were quantified using the Pierce quantitative colorimetric peptide assay. For a possible normalization and control of the chromatographic performance, the Thermo Scientific Pierce Peptide Retention Time Calibration Mixture consisting of 15 peptides was added to each sample.

#### LC-MS/MS analysis

The LC-MS/MS analysis was performed using high-performance liquid chromatography (HPLC) system, EASY-nLC™ 1000, connected to Q Exactive quadrupole Orbitrap mass spectrometer with a nanospray ion source.

Each sample, containing 1.00 μg of the Trypsin or Glu-C digested peptides in mobile phase A and 25 fmol of the retention time kit was injected at a flow rate of 300 nl/min and separated with a 132 min gradient of 5–22% ACN in 0.1% FA, followed by a 18 min gradient of 22–38% ACN in 0.1% FA. For the separation, a two-column setup was used, including the EASY-Spray analytical column (25 cm × 75 μm ID, particle size 2 μm, pore size 100 Å, PepMap C18) and the Acclaim pre-column (2 cm × 75 μm ID, particle size 3 μm, pore size 100 Å, PepMap C18). Each sample was measured in duplicate in a random order. The raw files obtained from the four measurements (Trypsin and Glu-C, replicate 1 and 2) of each sample were combined and evaluated using Proteome Discoverer targeting high confident peptides only.

The Q Exactive Plus system was operated in the positive data-dependent acquisition (DDA) mode to automatically switch between the full scan MS and MS/MS acquisition. For the peptide identification, full MS survey scan was performed in the Orbitrap detector. Fifteen data-dependent higher energy collision dissociation MS/MS scans were performed on the most intense precursors. The MS1 survey scans of the eluting peptides were executed with a resolution of 70,000, recording a window between m/z 400.0 and 1600.0. The automatic gain control (AGC) target was set to 1 × 10^6^ with an injecting time of 100 ms. The normalized collision energy (NCE) was set at 27.0% for all scans. The resolution of the data dependent MS2 scans was fixed at 17500 and the values for the AGC target and inject time were 5 × 10^5^ and 80 ms, respectively.

#### Identification of histone proteins and histone related post-translational modifications

The acquired MS/MS raw data files obtained from the combined randomized measurements were processed with Proteome Discoverer software, Version1.4 (Thermo Fisher), to identify the histone proteins including information regarding a number of unique peptides, sequence coverages and modifications.

The selection of spectra was based on the following settings: min precursor mass 350 Da; max precursor mass 5000 Da; s/n threshold 1.5. Parameters for Sequest HT searches were as follows: precursor mass tolerance 10 ppm; fragment mass tolerance 0.02 Da; depending on the sample type, trypsin or Glu-C was used as enzyme; 1 missed cleavage site; UniProt human database; dynamic modifications: acetyl (+42.011 Da; K), methyl (+14.016 Da; K, R), dimethyl (+28.031 Da; K, R), trimethyl (+42.047 Da; K, R), glygly (+114.043 Da; K) and oxidation (+15.995 Da; M, P) fixed modification: carbamidomethyl (+57.021 Da; C). The percolator was used for the processing node and the cutoff limit false discovery rate (FDR) value was set to 0.01. The selected spectra were used for the identification of histone proteins that were extracted and used for further analysis.

### Verification of the distinctly expressed H1.3 by IHC

The formalin fixed paraffin embedded (FFPE) PDAC specimens corresponding to fresh frozen preserved tissue analyzed with LC-MS/MS and normal pancreatic tissue, were sectioned and stained for the presence of Histone H1.3 antigen. Tissue sections with the omitting of the primary antibody were used as negative control. 4 μm tissue sections attached on a respective slide were deparaffinized and epitope retrieved using PT Link -PT 11730 (Dako, Agilent Technologies, Santa Clara, CA, United States) for 20 min at 97 °C in 1× EnVision ™ Flex retrieval solution, low pH (Dako, Agilent Technologies). The slides were then rinsed with Tris-buffered saline (25 mM Tris-HCl, 75 mM NaCl, 0.025% Triton-X, [pH 7.4]) and pretreated with 5% normal goat serum in dilution buffer (Tris-buffered saline with 1% BSA) for 1 hour at RT. The sections were then incubated overnight at 4 °C with 5 μg/ml anti- histone H1.3 polyclonal IgG (Abcam, Cambridge, MA, USA) recognizing N-terminal amino acids 7–33 of human histone H1.3. The endogenous peroxidase was blocked for 15 min at RT using 0.3% hydrogen peroxide and 1% methanol dissolved in TBS. The primary antibody was labeled with horseradish peroxidase (HRP) conjugated secondary antibody (Sigma-Aldrich), diluted 1:200 in dilution buffer. Diaminobenzidine (DAB) kit (Vector Laboratories Inc., Burlingame, CA, USA) was used as the substrate for colored visualization of the antigen and the nuclear contrast was achieved with hematoxylin counterstaining. The sections were then dehydrated, cleared with xylene and mounted with Pertex. The distribution of H1.3 in the tissue, the immunoreactivity, the overall staining intensity as well as the subcellular location, was evaluated by a practicing pathologist specialized in pancreatic cancer diagnostics, blinded to the clinical data. The staining was scored according to Giaginis et al. reviewed in Table 1 [18]. Finally, score sum of 2 was classified as low H1.3 expression and the score sum of ≥ 3 as high H1.3 expression. Representative images were taken at 10 and 20 x magnification using Olympus BX53 microscope.

**Table 1:** Evaluation of H1.3 immunohistochemistry [18]

Immunoreactivity	
	H1.3 + cells
0	0–4%
1	5–24%
2	25–49%
3	50–100%
Intensity	
0	negative
1	mild
2	intermediate
3	intense

### Analysis of H1.3 as a prognostic biomarker in PDAC

#### Clinical specimens

Clinical specimens were collected from patients with a suspected PDAC diagnosis, undergoing pancreaticoduodenectomy with a curative intent, at the department of Surgery, Skåne University Hospital in Lund, Sweden between 2000 and 2013. Resected tumors were histologically examined at the department of pathology, Skåne University Hospital in Lund to establish the diagnosis. Primary PDAC cases (*n* = 62) with a tumor located in caput pancreatis, were selected for the study and served as an external cohort for the evaluation of H1.3 as a prognostic biomarker. Pancreatic tissue obtained from patients with benign pancreatic disease (*n* = 10), were co-analyzed as a comparative control. The formalin fixed paraffin embedded (FFPE) specimens were acquired from the department of pathology, Skåne University Hospital in Lund for immunohistochemical analysis of H1.3, performed as reported above.

### Statistical analysis

The findings regarding histone profile, analyzed by LC-MS/MS, were assessed as presence or absence of the respective histone protein variant and analyzed as categorical data using Fisher’s exact test. In the analysis of H1.3 as a prognostic biomarker, the correlation between H1.3 expression and clinicopathological parameters was determined using the Mann-Whitney U test for continuous variables and Fisher’s exact test or χ^2^ for categorical variables. The Kaplan-Meier method was used to estimate the survival for patients with positive or negative H1.3 tumor expression. *P*-values estimating the differences between groups were calculated using the log-rank test. Clinical relevant confounding variables were identified from previously published studies including age, gender, tumor diameter, grading, lymph node metastasis, margin status and adjuvant chemotherapy. Adjustment for these confounding variables was made using the Cox proportional hazard method. A value of *p* < 0.05 was considered as statistically significant. STATA MP statistical package version 14.1 (StataCorp LP, College Station, TX) was used for the statistical analyses.

## Results

### Analysis of the histone profile in PDAC using LC-MS/MS

#### Profile of histone variants and histone related PTMs

Overall, it was possible to classify between 1281 and 2767 protein identifications of which 11 to 16 different histone variants were detected in individual samples. The number of histone protein identification was independent of the total number of protein identifications. For each detected histone protein subtype, the number of unique peptides as well as the total yield of high confidence peptides resulting from both Glu-C and trypsin digestion, was comparable in both experimental groups. Even though the sequence coverage was substantially improved by using additional digestion enzyme, the sequence coverages for the reported histone variants varied between 12.45% and 73.02%.

**Fig. 1:**
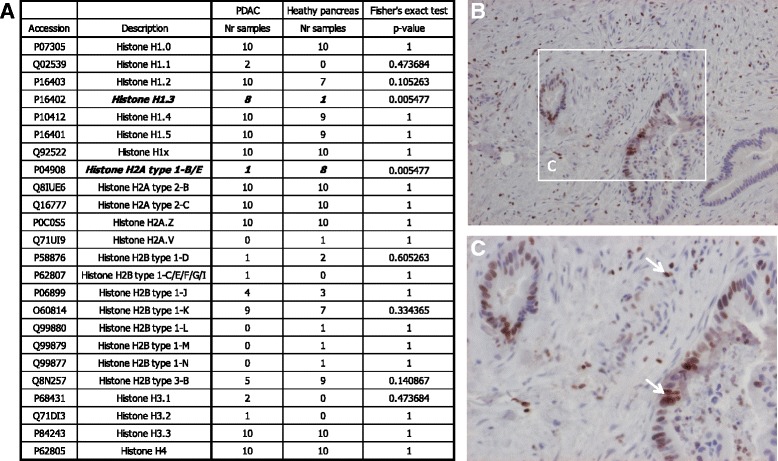
Profile of histone variants and H1.3 distribution in PDAC tissue. Histone variant profile is summarized in (A). Positive staining of H1.3 in PDAC is illustrated in (B) and (C). Positive H1.3 staining of tumor cells and TILs is indicated by the arrows. The images were magnified 10× (B) and 20× (C)

In total, we identified 24 variants of histone proteins, represented by at least one unique peptide sequence alignment, classified to the linker histone H1 family or core families comprising H2A, H2B, H3 and H4. Fourteen histone subtypes (58%) distributed among the five main histone families displayed the same pattern of frequency in both pancreatic cancer tissue and normal pancreas. The comprehensive histone profile is summarized in Fig. 1.

Altogether, we have identified seven H1 histone subtypes including H1.1-H1–5, H1.0 and H1x, where H1.3 was found significantly more frequent (*p* = 0.005) in PDAC material as compared to healthy control. H1.1 was present in 20% of the PDAC material, while absent in normal tissue. H1.0, H1.2, H1.4, H1.5 as well as H1x, were distinguished in the majority of the analyzed material.

The H2A family comprised totally five diverse subtypes, H2A1-B/E, H2A2.B H2A.C, H2A.Z and H2A.V. The abundance of H2A1-B/E was significantly lower (*p* = 0.005) in PDAC material. H2A.V was absent in all patient samples and found exclusively in 10% of normal pancreatic tissue. The H2A2.B, H2A.C and H2A.Z were identified in more than 90% in all of the tissue specimens.

Concerning all measured samples, the H2B histone family was represented by eight subtypes, identified as H2B1.D, H2B1.C/E/F/G/I, H2B1.J- H2B1.N and H2B3.B.

H3.1 and H3.2 were present distinctly in 20% and 10% of PDAC samples, respectively. H3.3 and H4 were identified in all analyzed samples.

**Fig. 2:**
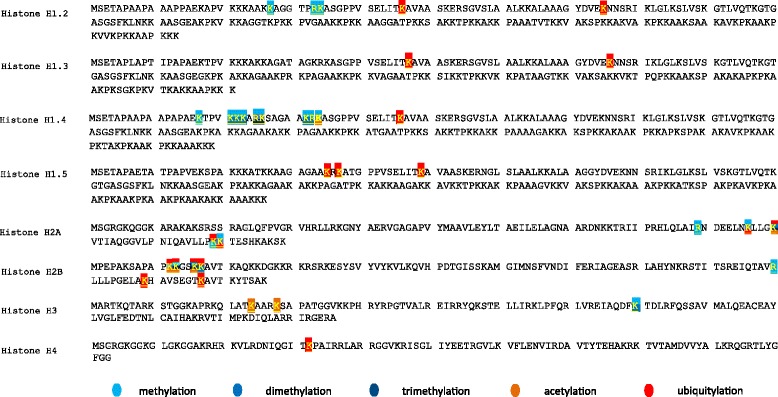
Distribution of histone-associated PTMs. Amino acid sequence alignments, representing the linker histone H1 variants and the main core histone families. The individual modified residues are indicated by the color of the annotated modifications

The detected peptides were investigated regarding possible dynamic PTMs. We have noted varied sporadic PTMs including acetylation (Ac), ubiquitination (Ub), methylation (Me), di- and trimethylation (Me2, Me3). The majority of the identified PTMs were annotated in a single sample within the respective group, showing a diffuse and inconsistent arrangement. PTMs presented in more than five samples in the individual groups (H2AR89Me, H2AK119Ub, H2AK120Ub, H2BR100Me, H2B109Ub, H3K80Me and H3K80Me2) revealed overlapping distribution pattern among the histone variants, resulting in a non-significant outcome. The complex array of the PTMs is summarized in Fig. 2.

#### Verification of the distinctly expressed H1.3

Immunohistochemistry was applied to verify the distinct expression pattern of linker histone variant H1.3 detected in PDAC tissue analyzed with LC-MS/MS (*n* = 10). Comprehensively, all investigated PDAC samples were positive for the H1.3 histone variant identified by a staining with the intensity ranging between mild and intense, assessed as a nuclear or membrane and cytoplasmic reaction. The H1.3 was identified in the 20–80% of tumor cells. H1.3 staining was also detected in tumor infiltrating lymphocytes (TILs) situated throughout the inflammatory stroma. Normal pancreatic tissues (*n* = 10) stained negative for H1.3. The representative pattern of H1.3 intra-tumor distribution is illustrated in Fig. 1.

### Analysis of H1.3 as a prognostic biomarker in an external PDAC cohort

#### Intratumor distribution of H1.3

**Fig. 3:**
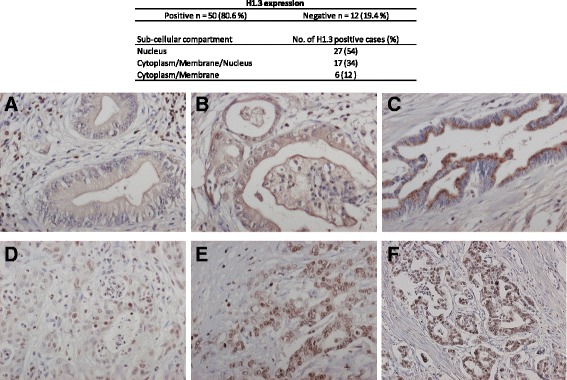
Subcellular expression of H1.3 in PDAC specimens. Membrane/cytosol staining of H1.3 in PDAC tumor cells with mild, intermediate and intense intensity is illustrated in (**a, b** and **c**), respectively. The nuclear staining of H1.3 in PDAC tumor cells with mild, intermediate and intense intensity is illustrated in (**d, e** and **f**), respectively. The images were magnified 20× (**c**)

The expression status of H1.3 in the PDAC specimens (*n* = 62) was evaluated using IHC. As presented in Fig. 3, 81% of the PDAC samples exhibited intratumor H1.3 expression, where nuclear reactivity was detected in the majority of the positively stained malignant cells (88%). In 34% of H1.3 positive cases, a nuclear, cytosolic and membrane reactivity was noted, while 12% of cases presented exclusively cytosolic and membrane reactivity. Lymphocytes infiltrating the tumor stroma stained positive for H1.3 in all PDAC specimens. Benign tissue stained negative for H1.3.

#### Expression of H1.3 correlates with poor prognosis in PDAC

As reported in Table 2, H1.3 expression was significantly associated with the age of the patient (*p* = 0.012). No significant correlations were shown between H1.3 expression and the additional clinicopathological factors including gender, tumor size, grade of differentiation, lymph node metastasis, resection margin status or adjuvant chemotherapy.

**Table 2:** The correlation between H1.3 expression and clinicopathological data in resected PDAC (*n* = 62)

		H1.3 expression		
	No. of patients (%)	Positive *n* = 50	Negative *n* = 12	*P*-value
Age (years), median [IQR]	67 [43–76]	66 [43–78]	73 [58–76]	0.012
Male gender	29 (47)	25 (50)	4 (33)	0.173
Tumor size (cm), median [IQR]	3 [0.3–8.5]	3 [1–8.5]	3 [0.3–4]	0.285
Poor differentiation	36 (58)	31 (62)	5 (42)	0.173
Lymph node metastasis	38 (61)	31 (62)	7 (58)	0.815
Resection margin status (R1)	18 (29)	14 (28)	4 (33)	0.721
Adjuvant chemotherapy	46 (74)	36 (72)	10 (83)	0.420

*IQR* interquartile range

**Fig. 4:**
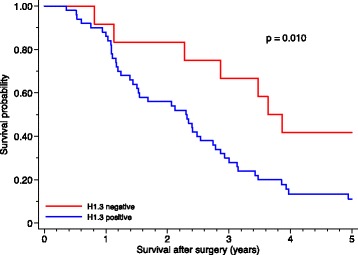
Kaplan-Meier survival curves for patients with positive or negative H1.3 expression

Kaplan-Meier analysis, reported in Fig. 4 revealed that H1.3 expression was associated with decreased median survival. The median survival of patients with negative H1.3 expression was estimated to 46 months with a 5-year survival of 42%. Patients with positive H1.3 expression showed a median survival of 28 months with a 5-year survival of 11% (*p* = 0.010).

Multivariate analysis indicated that the positive H1.3 expression was associated with a decreased survival, presented in Table 3.

**Table 3:** Multivariate Cox regression analysis (*n* = 62)

	Hazard ratio	95% CI	*P*-value
Unadjusted			
H1.3 expression (positive vs negative)	2.4	1.2–5.3	0.018
Adjusted^a^			
H1.3 expression (positive vs negative)	2. 6	1.1–6.1	0.029

*CI* confidence interval. ^a^Adjusted for age, gender, tumor size, differentiation, lymph node metastasis, margin status and adjuvant chemotherapy

## Discussion

Histone variants as chromatin remodeling proteins are emerging as important factors in cancer biology [9]. Thus the intratumor profiling of the distinct histone subtypes may lead to identification of interesting histone protein candidates, useful for the improvement of the disease management.

We performed a classical bottom-up MS analysis of PDAC tissues and normal pancreas biopsies to map the individual histone variants and histone related PTMs that could be correlated to chromatin dynamics in PDAC.

The profiling of histone variants revealed that H1.3, detected in the majority of patient samples and H2A1-B/E, primarily associated with the normal pancreatic tissue, displayed the opposite signatures of frequency with the overall accuracy of 95%. H1.3 expression in PDAC tissue was thereafter confirmed by IHC. H1.3 was thus considered as a possible PDAC specific histone variant candidate for further investigation. The immunohistochemical verification of H2A1-B/E was somewhat limited due to the high homology of the primary amino acid sequence of the H2A variants. However, MS based characterization of H2A histone family in ovarian cancer cells, revealed that the expression of the canonical histone variant H2A1-B/E was associated with undetectable levels [19], which we estimated, supports our findings.

The H1 linker histone family represents the most heterogeneous and functionally divergent group of histones among the highly conserved histone protein families. In tumorigenesis, the most relevant functional differences between the individual H1 subtypes are related to chromatin dynamics and transcriptional regulation. [20]. H1 linker histones consist of a highly conserved globular domain, variable N-terminal region and C-terminal domain (CTD), rich in positively charged lysine and arginine residues, that mediates both chromatin condensation and protein-protein interactions [21]. H1.3 was defined as a histone H1 subtype with an intermediate chromatin affinity that is associated with more relaxed and accessible chromatin conformation [22]. The results of global habitation studies demonstrated that H1.3 binds chromatin with significantly higher dynamics compared to the main H1 variants, thus the effect of H1.3 nucleosomal incorporation may be more pronounced at the specific binding sites [23]. According to a recent report, the CTD of histone H1.3 possesses the ability to recruit and interact with DNA methyltransferases, DNMT1 and DNMT3B, leading to methylation of CpG sites and subsequent gene silencing [24]. In cancer, a hypermethylation of CpG islands in promotor regions was described to correlate with transcriptional silencing of tumor suppressor genes [25] or with genes critical for the sensitivity to chemotherapy [26]. H1 was also shown to inhibit acetylation of H3 as well as methylation of nucleosomal H3K4 by interference with histone acetylase PCAF and histone-lysine N-methyltransferase SET7/9. Loss of histone acetylation as well as methylation of H3K4 represent events generally associated with a decreased transcriptional activity [24, 27, 28]. As recently reported, PTMs, H2AK119Ub and H2BK120Ub are involved in modulation of SET7/9 expression and regulation of H3K4Me2 and H3K9Me2 [29]. Moreover, methylation of individual CpG sites as well as low cellular levels of H3K4me2, H3K9Me2 and H3K18, were each reported as significant predictors of survival in PDAC [30, 31].

Our results indicate that H1.3 expression in PDAC tumors is associated with poor survival. Though, it remains possible that nucleosomal H1.3 may participate in the epigenetic regulation of gene repression in PDAC and by that contribute to the aggressiveness of the disease and poor prognosis.

The function of histone H1.3 subtype in pancreatic cancer is yet to be revealed and further investigations on this subject are necessary.

Interestingly, the positive H1.3 staining exhibited in tumor cells demonstrated, besides nuclear, also membrane and cytoplasmic distribution. Based on these results, we speculate that the biological role of H1.3 in PDAC may expand beyond the nuclear function. According to the findings from several reports, it appears that histones, in response to environmental stress, are frequently shuttled to the cell surface or cytoplasm where they act as signaling molecules [32]. Depending on the environmental conditions, histone proteins exposed to the extracellular matrix can function as basic ligands to negatively charged molecules, such as various proteoglycans, and regulate various cellular processes, including cell proliferation and matrix remodeling [33, 34]. The frequent cell proliferation associated with cancer development [35] may thus result in extensive transcription and synthesis of histone proteins. An overbalanced synthesis of nuclear proteins as histones may lead to cytoplasmic accumulation, as we observed in the IHC analysis of H1.3 in PDAC tissue.

Lymphocytes infiltrating the tumor and signaling pathways related to the immune system are frequently observed in the immunogenic subclass of pancreatic cancer [5]. Consistent with the findings of the present study, the infiltrating lymphocytes are found prevalently in the stromal compartment as a functional part of the tumor microenvironment [36]. Further investigation is however required to understand the possible contribution of TILs expressing high levels of H1.3 to the development and progression of pancreatic cancer.

## Conclusions

We found that the expression of H1.3 in tumor cells provides prognostic information in patients with PDAC. Our results suggest that H1.3 may serve as a novel epigenetic biomarker for the prediction of clinical outcome after surgical resection. The intratumor histone profile, especially the distinct histone subtypes, may also contribute to the increased understanding of pancreatic tumor biology and should be considered for further investigation aiming to improve the clinical management of PDAC.
